# Patients’ Engagement With “Sweet Talk” – A Text Messaging Support System for Young People With Diabetes

**DOI:** 10.2196/jmir.962

**Published:** 2008-06-30

**Authors:** Victoria Louise Franklin, Alexandra Greene, Annalu Waller, Stephen Alan Greene, Claudia Pagliari

**Affiliations:** ^4^Division of Clinical and Community Health Sciences (GP Section)University of EdinburghEdinburghUK; ^3^School of ComputingUniversity of DundeeDundeeUK; ^2^Health Service Research UnitUniversity of AberdeenAberdeenUK; ^1^Maternal and Child Health SciencesNinewells Hospital and Medical SchoolDundeeUK

**Keywords:** Diabetes mellitus, adolescent, eHealth, social support, text messaging

## Abstract

**Background:**

Guidelines for optimizing type 1 diabetes in young people advocate intensive insulin therapy coupled with personal support from the health care team. “Sweet Talk” is a novel intervention designed to support patients between clinic visits using text messages sent to a mobile phone. Scheduled messages are tailored to patient profiles and diabetes self-management goals, and generic messages include topical “newsletters” and anonymized tips from other participants. The system also allows patients to submit data and questions to the diabetes care team.

**Objectives:**

The aim was to explore how patients with type 1 diabetes interact with the Sweet Talk system in order to understand its utility to this user group.

**Methods:**

Subjects were 64 young people with diabetes who were participating in the intervention arms of a randomized controlled trial. All text messages submitted to Sweet Talk during a 12-month period were recorded. Messaging patterns and content were analyzed using mixed quantitative and qualitative methods.

**Results:**

Patients submitted 1180 messages during the observation period (mean 18.4, median 6). Messaging frequency ranged widely between participants (0-240) with a subset of 5 high users contributing 52% of the total. Patients’ clinical and sociodemographic characteristics were not associated with total messaging frequency, although girls sent significantly more messages unrelated to diabetes than did boys (*P* = .002). The content of patients’ messages fell into 8 main categories: blood glucose readings, diabetes questions, diabetes information, personal health administration, social messages, technical messages, message errors, and message responses. Unprompted submission of blood glucose values was the most frequent incoming message type (35% of total). Responses to requests for personal experiences and tips generated 40% of all the incoming messages, while topical news items also generated good responses. Patients also used the service to ask questions, submit information about their self-management, and order supplies. No patients nominated supporters to receive text messages about their self-management goals. Another option that was not used was the birthday reminder service.

**Conclusions:**

Automated, scheduled text messaging successfully engaged young people with diabetes. While the system was primarily designed to provide “push” support to patients, submission of clinical data and queries illustrates that it was seen as a trusted medium for communicating with care providers. Responses to the newsletters and submission of personal experiences and tips for circulation to other participants also illustrate the potential value of such interventions for establishing a sense of community. Although participants submitted relatively few messages, positive responses to the system suggest that most derived passive support from reading the messages. The Sweet Talk system could be readily adapted to suit other chronic disease models and age groups, and the results of this study may help to inform the design of future text message support interventions.

## Introduction

Diabetes is a condition requiring considerable self-management of diet, exercise, and medication use, and this can be challenging for children and adolescents. Recent guidelines on the management of type 1 diabetes recommend that young people should be offered intensive insulin therapy in conjunction with a package of care including emotional and behavioral support [1,2]. However, increasing the frequency of direct clinical contact is costly, and young people can fail to engage with conventional group-based support activities [3,4].

Emerging information and communications technologies have considerable potential to aid patients with long-term conditions, and young people with diabetes report using many of these to serve their information and support needs [5]. For example, analysis of messages submitted to online diabetes forums suggests that adolescents use these in order to obtain social support, information, advice, and shared experience [6].

Text messaging via mobile phones has become an integral component of teenage culture in many parts of the world, providing an inexpensive, portable, and widely available form of communication [7]. Over a third of US teenagers and 80% of UK teenagers reported using text messaging in national surveys published in 2005, and these figures are undoubtedly increasing [7,8]. The medium is increasingly being used to deliver health care information, reminders, and lifestyle interventions and has obvious potential to engage young people with diabetes [9].

For these reasons, we developed the “Sweet Talk” system, which delivers tailored motivational messages to young people with type 1 diabetes using text messaging. In a randomized controlled trial, this was shown to have positive effects on diabetes control, self-efficacy, and adherence, and user questionnaires indicated high patient acceptability [10]. However, understanding how such complex interventions work requires an appreciation of how they are adopted and used by their intended targets. While the Sweet Talk system was primarily designed to deliver passive “push” support to patients, its capacity to allow them to submit or reply to messages presented an opportunity to explore these issues. This paper describes an analysis of patients’ interactions with Sweet Talk that sought to inform our understanding of how users integrate such systems into their daily lives, the elements that they engage with the most, and any unexpected uses.

## Methods

### Description of the Sweet Talk System

**Figure 1:**
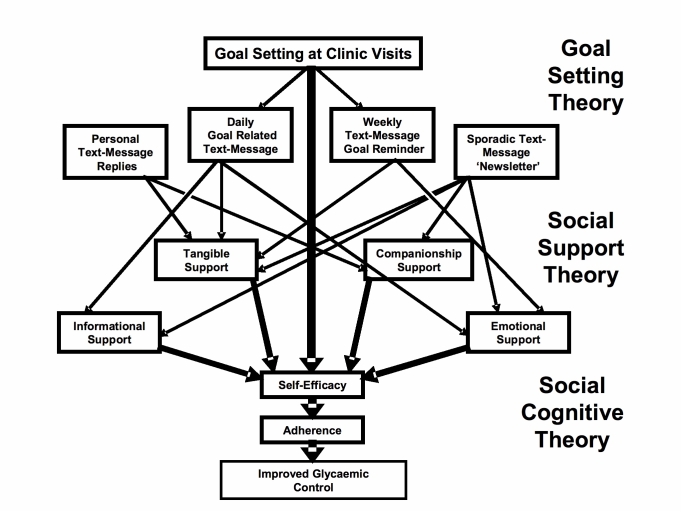
Theoretical basis of the Sweet Talk intervention

**Figure 2:**
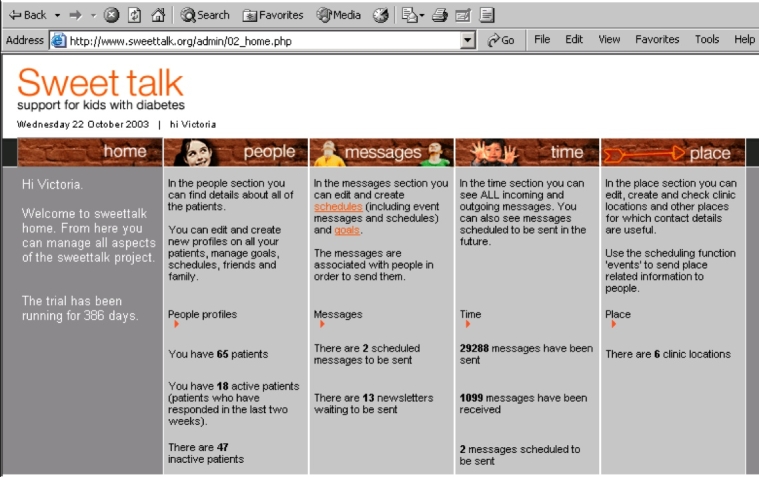
Screenshot of Sweet Talk

Sweet Talk is a novel intervention for supporting young people with type 1 diabetes through text messaging. The intervention is informed by social cognitive theory, which states that health behaviors are influenced by self-efficacy, or the belief in one’s ability to perform actions that will influence outcomes [11], which, in turn, is influenced by goal setting and social support [12,13]. The system was designed to deliver a unique form of push support [14], in contrast to conventional support groups and websites where users have to actively access a site to read messages [15], thus favoring motivated patients and potentially enhancing health inequalities [9,16,17]. The system contains a database of text messages, including information, tips, and reminders categorized according to the main diabetes self-management tasks of insulin injections, blood glucose testing, healthy eating, and exercise. Messages are automatically scheduled based on patient profiles (age, gender, and treatment regimen) and personal diabetes self-management goals created at each clinic visit (healthy eating, exercise, insulin injections, and blood glucose testing). Such personalization appears fundamental to behavioral support interventions [18-20]. Patients receive a weekly text message reminder of their personal goal and a daily text message from the database, thus receiving either one or two messages daily. In addition, patients receive occasional text “newsletters” relating to topical issues and asking questions about their own diabetes self-management routine. They are encouraged to send in messages containing information or questions related to their diabetes self-management. Patients’ ideas and responses that are felt likely to be of general interest are forwarded anonymously to the whole group in order to develop a sense of community among the participants while avoiding the risks of unmoderated peer-to-peer networks, such as the sharing of health-harming practices and text bullying [7,21,22]. The different message types are explained further in a previous paper describing the development of the system [14]. Since Sweet Talk aims to motivate effective self-care, it may also be regarded as a type of “persuasive technology” [23]. Figure 1 represents the theoretical model of the intervention, and a screenshot of the Web-based interface is shown in Figure 2.

### Subjects and Procedures

The subjects were 64 boys and girls aged 8-18 years with type 1 diabetes participating in the intervention arms (Sweet Talk plus conventional therapy n = 33; Sweet Talk plus intensive therapy n = 31) of a three-arm clinical trial during a 12-month period between October 2002 and March 2004.

**Figure 3:**
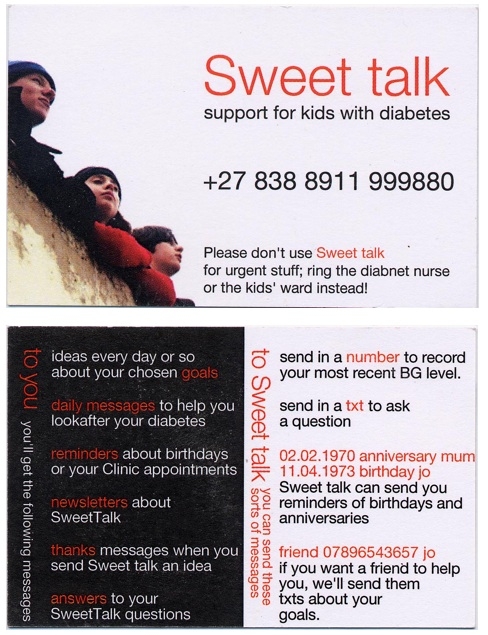
Sweet Talk information card

Written informed consent was obtained from patients and their families, and the study was approved by The Tayside Committee on Medical Research Ethics. Participating patients received a pay-as-you-go mobile phone and a £10 phone card, and incoming text messages to the Sweet Talk system were free of charge. Mobile phones could also be used for personal use. Patients allocated to Sweet Talk were given an information card highlighting the messages that they could expect to receive and suggestions of how they could use the system (Figure 3).

### Quantitative Methodology

All of the text messages sent to and from the Sweet Talk system were recorded over the 12 months of the study. This produced observational data on messaging patterns, which could be triangulated with patient clinical and demographic data, as well as message transcripts. Post-hoc analyses for associations between message content and demographic variables were undertaken using chi-squaretests for categorical variables and analysis of variance (ANOVA) for continuous variables.

### Qualitative Methodology

Message transcripts were analyzed by VF using the constant comparative method in order to generate descriptive themes [24]. This process was facilitated by Nvivo textual analysis software (QSR International, Doncaster, Australia). AG independently analyzed 10% of the messages in order to validate the themes identified by the first rater, and inconsistencies were resolved through discussion. Further verification was achieved through team-based review of identified themes and representative raw data by VF, AG, and CP. Transcripts were then content-analyzed by VF according to these thematic categories [25].

## Results

### Frequency of Patient Interactions With the Text Messaging Service

**Figure 4:**
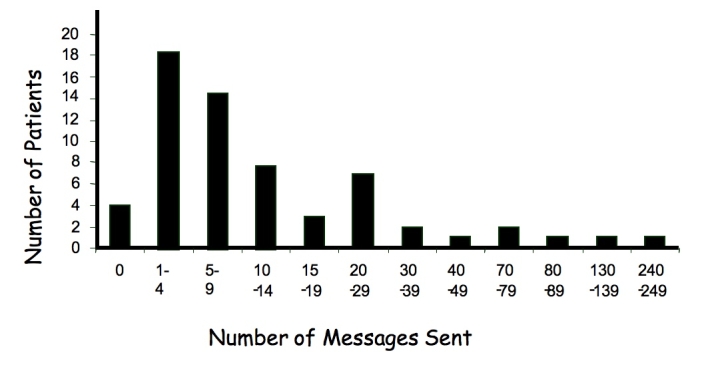
Number of messages sent to Sweet Talk during the 12-month study

All but 4 of the 64 patients allocated to the Sweet Talk intervention submitted one or more text messages during the 12 months of the study. A total of 1180 messages were submitted, representing an average of 18.4 messages per patient. However, total messaging varied widely between individuals, from 0 to 240 (median 6), and the distribution was skewed by 5 patients who contributed 52% (614/1180) of the messages (Figure 4). A significant proportion of these messages were from 2 boys who sent in very regular blood glucose readings, comprising 338 of the total 1180 messages received (29%).

No participants took the opportunity to use the birthday reminder service or to nominate family or friends to receive patients’ goal reminders so that they could act as personal supporters—two options offered on the information card (see Figure 3).

### Association Between Messaging and Patient Characteristics

There were no associations between the total number of messages submitted to Sweet Talk and patients’ social or clinical demographics, including age, gender, duration of diabetes, insulin regimen, HbA_1c_ (glycosylated hemoglobin), or social deprivation score, all determined using ANOVA for the continuous variables and chi-square tests for the categorical variables (*P* > .05). Post-hoc analyses for associations between message content and demographic variables identified one significant association with gender: females sent significantly more messages containing information and questions unrelated to diabetes (females: mean 1.53 ± 2.51; males: 0.09 ± 0.30; *P* = .002).

Patients who had expressed positive attitudes toward Sweet Talk in a user survey [10] were no more likely to have submitted messages to the system than those who had not. Patients sending messages to Sweet Talk received a higher number of personalized responses (*r* = .521, *P* = .01).

### Text Message Themes

The content of the text messages that patients sent to Sweet Talk fell into 8 broad thematic categories covering blood glucose readings, diabetes questions, diabetes information, personal health administration, social aspects, technical messages, message errors, and message responses. Illustrative text messages are shown in Table 1. The total exceeds 1180 because 77 messages were coded into more than one category.

**Table 1:** Main themes from patient-submitted text messages

Theme	No. (%)	Example
Blood glucose tests	418/1180 (35)	“This morning my blood was sitting at 5.7”
Diabetes questions	74/1180 (6)	“Is it ok 2 do nova rapid just before or after a lantus injection?”
Diabetes information	50/1180 (4)	“I hav managed 2 change my injection site 4 a few days now! =)”
Personal health administration	63/1180 (5)	“Could i have a onetouch ultra meter because went through the wash on holiday”
Social messages	75/1180 (6)	“I slept over at 2 friends houses.it was great,it was my first time”
Technical messages	86/1180 (7)	“Its hard 2 send txts bak 2 u cuz this fom dosnt get coverage.”
Message errors	19/1180 (2)	“Nyt Nyt Dad”
Responses to Sweet Talk messages	472/1180 (40)	“More hard coz of parties & sleepovers” (Txt in and let us know what ur doing in the holidays - do holidays make it easier or more difficult to control ur blood sugars?)”

### Blood Glucose Testing

Messages containing blood glucose values accounted for 35% of all messages (418/1180). Of these, 56% (232) followed the advice to submit blood glucose values alone (see Figure 3), while the remainder incorporated these values within text. Two boys contributed to 81% of the total blood glucose text messages sent.

### Diabetes Questions

Messages containing questions related to some aspect of diabetes self-management made up 6% (74/1180) of all messages. Sweet Talk appeared to provide an opportunity for obtaining information between clinic visits and to send questions that patients may have found difficult to ask in a clinical setting (eg, “Cld DiaBT’s get their belly pierced”). Text messages containing diabetes questions were further categorized into topic themes, as illustrated in Table 2.

**Table 2:** Examples of diabetes questions submitted by patients

Topic	No. (%)	Example
Blood glucose	16/74 (22)	“my bg's hav bin runnin a bit higher than usual for the past couple of weeks cos of exams. Any tips on how i can get them back to normal?”
Exercise	4/74 (5)	“Im finding it difficult 2 find the time 2 exercise with my exams being so near what should i do?”
Insulin	8/74(11)	“Is it ok 2 do nova rapid just before or after a lantus injection?”
Diet	7/74 (9)	“What can i have to eat when my Friends are having sweets?”
Pump	11/74 (15)	“Wen ur in the bath or shower, wot hapens if anythng gets in2 the infusion set even with the clip on?”
Carb counting	5/74 (7)	“Hi quick question. Does popcorn count as Carbs? What effectwill it hav on my bg's?”
HbA_1c_	2/74 (3)	“Could u tell me my hb1ac result that i was tested 4 on tuesday at montrose?”
Goals	1/74 (1)	“Can you tell of my goal because i cant remember what i wrote on the sheet”
Emergency	1/74 (1)	“I got ketones…. bloods r up …. HELP!”
Other	19/74 (26)	“Cld DiaBT’s get their belly pierced”

### Diabetes Information

Messages containing information about a patient’s own diabetes self-management or health status accounted for 4% (50/1180) of all messages (eg, “I hav managed 2 change my injection site 4 a few days now! =)”). Sweet Talk also provided an outlet for expressing frustration with their diabetes. One “emergency” message was received: “I got ketones bloods r up HELP.” This message was sent despite clear instructions on the information card that the Sweet Talk system was not intended for this use and that patients should continue to use our emergency help line. Telephone follow-up revealed that the patient knew this but simply wanted to know what would happen if he sent a message of this kind.

### Personal Health Administration

Patients were encouraged to use the Sweet Talk system as an easy method of contacting the diabetes team with any requests. Of the total messages, 5% (59/1180) contained requests for supplies such as insulin pump consumables, blood glucose meters, and insulin travel authorization letters (eg, “Could I have new meter because it went through the wash on holiday”) and requests for information about clinic appointments (eg, “Hi, can you please tell me when my next clinic appointment is. Thank you”).

### Social Messages

Messages of a social nature made up 6% (75/1180) of patients’ incoming messages. Although not directly related to diabetes, these messages provide insight into how patients integrated the system into their daily lives and its value as a source of social support (eg, “Just ate an ice-cream and done a dual wave. Off to colosseum!” and “Happy xmas 2 every1 at 9wels ”). Post-hoc analyses for associations between message content and demographic variables identified gender differences: females sent significantly more messages containing information and questions unrelated to diabetes (females: mean 1.53 ± 2.51; males: 0.09 ± 0.30; *P* = .002).

### Technical Messages

Messages about technical aspects of the Sweet Talk system accounted for 7% (86/1180). Of these, most were related to difficulties with message transmission and cost of the messages (n = 55). A further 18 messages indicated problems with the content of the Sweet Talk messages, highlighting failures in message personalization or not understanding the messages (eg, “I keep getting messages about injections but I’m on the pump”).

### Message Errors

Of the total messages, 19 (2%) appeared to have been sent to Sweet Talk in error (eg, “Nyt nyt Dad”).

### Responses to System-Generated Messages

Messages that were sent by patients in direct response to a Sweet Talk text message made up 40% (472/1180, Table 3). Of these, the sporadic text message newsletters generated the most responses (40%, 190/472). For example, one message that asked patients what symptoms they got when their blood sugars were running high provoked responses such as “I get thirsty and a dry throat when I’m high. I also can get a bit moody.” The four newsletters that triggered the greatest flurry of responses had updates on diabetes research, raised the issue of a chocolate manufacturer offering tokens for sports equipment, and reported about a film star and a soap opera character with diabetes. The remaining messages were in response to the daily scheduled messages (30%, 142/472), personal messages (25%, 118/472), and the weekly goal reminder (5%, 22/472). There was a significant correlation between the number of messages patients sent to Sweet Talk and the number of individual response messages they received (*r* = .521, *P* = .01).

**Table 3:** Patient responses to Sweet Talk system messaging

Type of Message	Number of Patient Messages	System Message	Patient Message
Scheduled	142	Have u tested today?	“Yes i have been 2.9 4.5 & 5.5”
Goal reminder	22	ur goal is 2 eat less sugary things 2 get ur bloods down!	“I no i am tryin’”
Newsletter	190	Txt in and let us know what ur doing in the holidays - do holidays make it easier or more difficult to control ur blood sugars?	“More hard coz of parties & sleepovers”
Responsive mode	118	Re: question about infusion set – the cannula is self sealing, so with or without clip nothing can get in.	Question: “Wen ur in the bath or shower, wot happens if anything gets in2 the infusion set even with the clip on?” Response: “Really? That’s good, its been at the bak of my mind 4 ages!”

## Discussion

### Principal Findings

While the primary intended function of Sweet Talk was to deliver passive support to patients, most participants in this study took the opportunity to submit messages to the system. Analysis of these messages has provided insight into the ways users may adapt text messaging interventions to best serve their needs. Although average messaging frequency was low, there was wide variation among participants, with most messages submitted by 5 power users. No associations were found between total messaging frequency and clinical or psychosocial measures. The content of patients’ messages fell into 8 broad categories covering submission of blood glucose readings, questions about diabetes treatment or lifestyle, information about diabetes self-management, personal health administration such as supply re-ordering, social messages, technical messages, messages sent in error, and responses triggered by a scheduled Sweet Talk message. Unprompted submission of blood glucose readings was most common, followed by messages submitted in response to a system-generated message. Of the latter, those suggesting that patients share tips and frustrations about diabetes self-management generated the most responses. Diabetes news items also stimulated many responses. Females sent significantly more text messages of a social nature, unrelated to diabetes, than did males. No participants took the opportunity to nominate family or peer supporters to receive their goal messages or used the birthday reminder function.

### Limitations

While the generalizability of the results is limited by the fact that only 5 users accounted for the majority of the messages, most participants interacted with the system during the study. This is consistent with observations of diabetes chat rooms, where only a minority of users post messages but the remaining lurkers read and benefit from other peoples’ messages [17,26]. In a user satisfaction questionnaire, reported separately, most participants indicated that Sweet Talk had helped them to look after their diabetes and that they wished to continue receiving messages at the end of the study period [10].

The lack of association between messaging frequency and clinical or psychosocial measures may reflect our choice of scoring systems, which were largely diabetes centered. Assessing personality measures such as neuroticism, extraversion, openness, agreeableness, and conscientiousness [27] may have revealed important associations and should be considered in future studies assessing the uptake and use of such interventions.

### Implications for Practice

The formative data generated by this study have helped to further our understanding of the fit of this technology with users’ needs, to challenge our pre-existing ideas about how it might support young people, and to generate ideas for refining the service.

Patients’ interactions with the system suggest that many valued the opportunity to engage in reciprocal communication, although not all participants chose to take advantage of this. Patients who more frequently submitted questions to the system inevitably received more individual responses, illustrating how motivated patients may obtain more personalized services despite efforts to design equitable technology-based support interventions.

Submission of blood glucose readings was the most common type of message, supporting results of studies indicating the potential for remote disease monitoring [28]. Using Sweet Talk to request supplies and enquire about appointments also has potential to increase efficiency through avoiding the telephone tag that can occur when health professionals and patients try to communicate between clinic visits [29]. The volume of text messages sent by patients over the year of the study was low, and minimal health professional time was required for correspondence with patients. This is consistent with studies of email consulting, which did not show the expected unmanageable burden of correspondence [30].

Newsletters containing information about topical aspects of diabetes or reports about public figures with the condition may have stimulated responses through reinforcing the sense of community and boosting self-esteem through identification with respected role models. Studies of disease-specific websites and chat rooms indicate that it is the information and companionship components that are most valued by patients [17,26].

Sweet Talk appears to have provided a forum for patients to ask personal or embarrassing questions that they may have felt unable to ask at a clinic visit. This is compatible with research demonstrating the value of computer-based interventions for encouraging disclosure of sensitive information, such as mental health problems [31].

Females’ greater use of the system for social messaging accords with studies of mobile phone and chat room use [6,17,32]. However, social messaging represented far fewer interactions in this study (4%) than in a previous telephone support study by our group, in which it accounted for the majority of talk time [33], possibly reflecting teenagers’ preference for verbal over written communication for social interaction [8].

While ongoing parental and peer support for diabetes self-management is important for optimizing glycemic control in adolescence [34-36], none of the participants in this study took the opportunity to nominate family or friends to receive messages related to their diabetes goals, which contrasts with results in other areas such as smoking cessation [37]. This may have been due to inadequate advertisement or explanation of the concept, lack of interest, or a concern that it would place patients under unhelpful pressure. A previous study in which children’s blood glucose readings were sent to a parent’s mobile phone showed that while this promoted a sense of reassurance in some children, in others it generated a feeling of surveillance and reduced their sense of personal control [38].

### Future Research

Our qualitative and usage data illustrate both individual differences in patients’ propensity to interact with the system and the multiple potential utilities that such complex interventions may provide. Further research to explore patterns of use in different age and clinical groups would be valuable, as would studies of the personal and contextual factors influencing the adoption of such technology.

Inviting patients to join the editorial board of similar text messaging interventions will help to ensure the appropriateness of message content and delivery schedules as well as identify users’ expectations for reciprocal messaging and the likely implications for practitioner time.
